# Batch-fabricated full glassy carbon fibers for real-time tonic and phasic dopamine detection

**DOI:** 10.3389/fbioe.2025.1543882

**Published:** 2025-02-28

**Authors:** Umisha Siwakoti, May Yoon Pwint, Austin M. Broussard, Daniel R. Rivera, X. Tracy Cui, Elisa Castagnola

**Affiliations:** ^1^ Department of Biomedical Engineering, Louisiana Tech University, Ruston, LA, United States; ^2^ Department of Bioengineering, University of Pittsburgh, Pittsburgh, PA, United States; ^3^ Center for Neural Basis of Cognition, University of Pittsburgh, Pittsburgh, PA, United States; ^4^ McGowan Institute for Regenerative Medicine, University of Pittsburgh, Pittsburgh, PA, United States; ^5^ Institute for Micromanufacturing, Louisiana Tech University, Ruston, LA, United States

**Keywords:** glassy carbon fibers, microelectrodes, dopamine, fast scan cyclic voltammetry (FSCV), square wave voltammetry (SWV)

## Abstract

Dopamine (DA) is a critical neurotransmitter that is key in regulating motor functions, motivation, and reward-related behavior. Measuring both tonic (baseline, steady-state) and phasic (rapid, burst-like) DA release is essential for elucidating the mechanisms underlying neurological disorders, such as schizophrenia and Parkinson’s disease, which are associated with dysregulated tonic and phasic DA signaling. Carbon fiber microelectrodes (CFEs) are considered the gold standard for measuring rapid neurotransmitter changes due to their small size (5–10 µm), biocompatibility, flexibility, and excellent electrochemical properties. However, achieving consistent results and large-scale production of CFE arrays through manual fabrication poses significant challenges. We previously developed flexible glassy carbon (GC) microelectrode arrays (MEAs) and GC fiber-like MEAs (GCF MEAs) for neurotransmitter detection and electrophysiology recording. We also demonstrated the feasibility of fabricating GC MEA with both GC electrodes and interconnects made from a single homogeneous material, eliminating the need for metal interconnections and addressing related concerns about electrical and mechanical stability under prolonged electrochemical cycling. Building on our prior experience, we now present a double-etching microfabrication technique for the batch production of 10 μm × 10 µm full GC fibers (fGCFs) and fGCF arrays, composed entirely of homogeneous GC material. This process uses a 2 µm-thick low-stress silicon nitride as the bottom insulator layer for the fGCFs. The effectiveness of the fabrication process was validated through scanning electron microscophy (SEM) and energy dispersive X-ray spectroscopy (EDS) elemental analyses, which confirmed the uniformity of the Si₃N₄ insulation layer and ensured the overall integrity of the fGCFs. Using finite element analysis, we optimized the fGCF form factor to achieve self-penetration up to 3 mm into the mouse striatum without additional support. The electrochemical characterization of fGCFs demonstrated high electrical conductivity and a wide electrochemical window. The ability of fGCFs to detect phasic and tonic DA release was confirmed using fast scan cyclic voltammetry (FSCV) and square wave voltammetry (SWV), respectively, both *in vitro* and *in vivo*. With their high sensitivity for phasic and tonic DA detection, combined with a scalable fabrication process and self-supporting insertion capability, fGCFs are promising sensors that offer enhanced practicality for comprehensive DA monitoring.

## 1 Introduction

Dopamine (DA) is an important neurotransmitter involved in regulating motor functions, motivation, and reward-related behavior (Baik, 2013; Speranza et al., 2021; Bressan and Crippa, 2005; Arias-Carrión and Pöppel, 2007; Ungerstedt et al., 1982). DA release occurs on multiple timescales, i.e., phasic and tonic release (Grace, 1991; Grace, 2000). Phasic release results from rapid burst-firing of neurons, leading to brief, high-concentration DA spikes in the synaptic cleft, important for signaling specific events or stimuli (Grace and Bunney, 1984; Cohen et al., 2012). In contrast, tonic release reflects the slower, continuous firing of neurons, which maintains extracellular DA basal levels through extra synaptic diffusion (Grace and Bunney, 1984; Grace, 2016; Goto et al., 2007). Dysregulation in the balance of phasic and tonic DA signaling has been implicated in severe neurological disorders, including schizophrenia, Parkinson’s disease and depression (Grace, 2016; Venton and Wightman, 2003; Sonnenschein et al., 2020). Thus, accurate measurement of both phasic and tonic DA release is essential for understanding normal brain function and the pathophysiology of these disorders.

For phasic DA detection, fast scan cyclic voltammetry (FSCV) is the current gold standard (Puthongkham and Venton, 2020; Rafi and Zestos, 2021; Venton and Cao, 2020; Robinson et al., 2003; Castagnola et al., 2020; Banerjee et al., 2020). With sub-second temporal resolution, FSCV captures rapid neurotransmitter dynamics in synaptic release events (Puthongkham and Venton, 2020; Roberts and Sombers, 2018). By using a fast scan rate of ∼400 V/s, FSCV measures the sharp changes in DA concentration by sweeping the potential across a window where DA oxidation and reduction occur (Puthongkham and Venton, 2020; Roberts and Sombers, 2018). However, FSCV relies on background subtraction techniques, making it unsuitable for detecting slower, tonic DA release (Taylor et al., 2019; Rusheen et al., 2020).

To detect tonic DA levels, different methods have been employed, including 1) microdialysis, 2) imaging techniques, and 3) electrochemical methods. Microdialysis has high chemical specificity and can measure DA concentrations at nanomolar levels (Chauhan et al., 2020). However, its low spatial and temporal resolution, along with the large probe size, can cause inflammatory responses, compromising sampling accuracy (Tan et al., 2021; Wonnenberg and Zestos, 2020). Imaging techniques such as functional MRI (fMRI) and positron emission tomography (PET) offer non-invasive insights and are well-established in clinical research. However, these methods are limited by poor spatial resolution (>1 mm), inability to directly measure tonic DA levels, and high operational costs (Rusheen et al., 2020; Tan et al., 2021). Genetically encoded fluorescent sensors (GEFS) are also used for neurotransmitter detection due to their high selectivity, cell specificity, and excellent spatiotemporal resolution (Wang et al., 2018). However, they require genetic modification of the cells and are optimized to measure changes in concentration, rather than basal level (Sun et al., 2018; Labouesse et al., 2020). Additionally, limited light penetration in biological tissues restricts the effective imaging depth, making it difficult to use GEFS in deep-brain regions (Xiao et al., 2023; Zhang et al., 2023).

Given these limitations, due to the recognized superior spatial and temporal resolution of electrochemical techniques (Rusheen et al., 2020; Tan et al., 2021; Liv, 2024a; Liv, 2023), several voltammetric methods have been modified and optimized for the detection of tonic DA levels, including differential normal pulse voltammetry (Gonon et al., 1984; Marcus et al., 2001), fast-scan controlled-adsorption voltammetry (FSCAV) (Atcherley et al., 2013; Atcherley et al., 2015), charge-balancing multiple waveform FSCV (CBM-FSCV) (Oh et al., 2016), convolution-based FSCV (Johnson et al., 2018), and multiple cyclic square wave voltammetry (M-CSWV) (Oh et al., 2018; Barath et al., 2020). These techniques have been successfully applied *in vivo* and demonstrated successful detection of extracellular tonic DA concentrations in a ∼50–100 nM range. We recently optimized a square wave voltammetry (SWV) waveform for the detection of basal DA levels in rodent brains, both at PEDOT/CNT-coated (Taylor et al., 2019; Castagnola et al., 2022) and bare GC electrodes (Castagnola et al., 2022; Castagnola et al., 2024), obtaining DA measurement in the same concentration range. SWV is a pulse voltammetry method specifically suited for the measurements of resting analyte concentrations (Taylor et al., 2019; Castagnola et al., 2022; Bard et al., 2022). SWV presents high sensitivity and is very effective for the isolation of faradaic currents—arising from redox reactions of electroactive analytes—from capacitive charging currents, thereby enabling precise detection of basal DA levels (Taylor et al., 2019; Castagnola et al., 2022).

Carbon fiber electrodes (CFEs) are the most commonly used electrodes for FSCV due to their small size, chemical stability, fast electron transfer kinetics, and low background currents (Huffman and Venton, 2009; Kakhki, 2019). However, a major drawback of CFEs and CFE arrays lies in their manual fabrication process, which limits scalability. To address this limitation, photolithography-based techniques have been developed for the batch fabrication of carbon-based microelectrode arrays (MEAs) (Castagnola et al., 2022; Castagnola et al., 2023; Castagnola et al., 2021a; Wu et al., 2023). This approach enables the production of high-density, multi-channel arrays with consistent electrode properties. However, current procedures for implanting planar MEA require the use of a tungsten wire, which is anchored to the MEA shank via an anchor hole to facilitate precise placement in rodent brains (Castagnola et al., 2022; Castagnola et al., 2023; Wu et al., 2023). Although the guide wire is removed immediately after implantation, its use introduces insertion trauma, that can lead to tissue damage and delay healing. To address this issue, we have recently fabricated glassy carbon fiber-like MEAs (GCF MEAs) using photolithography (Castagnola et al., 2024). These GCF MEAs incorporate fiber-like GC electrodes with small cross-sections, facilitating self-insertion into brain tissue without additional support. Compared to traditional CFEs, these GCF MEAs demonstrated superior electrochemical performance, detecting both tonic and phasic DA concentrations, as well as recording single-unit activity (Castagnola et al., 2024).

One potential concern for the chronic use of these “hybrid” MEAs, which combine GC electrodes with metal interconnections, is the mechanical mismatch at the carbon-metal interface, which can result in mechanical failure during prolonged electrical stimulation. To overcome this challenge, our group has recently developed GC-MEAs (GC-MEAs) with GC electrodes and interconnections, using two fabrication approaches: *double pattern transfer* (Faul et al., 2024; Nimbalkar et al., 2018) and *double dry etching* (Faul et al., 2024). While the double pattern transfer method demonstrated effectiveness, its complexity limited miniaturization and scalability. In contrast, the double dry etching technique offered a more streamlined process, showing promise for producing miniaturized devices (Faul et al., 2024). Leveraging the potential for miniaturization of this fabrication method (Faul et al., 2024), we now present self-inserting full GC fibers (fGCFs) and fGCF arrays, eliminating the need for metal interconnection and tungsten wire as a guide, offering an interesting solution for high-performance, minimally invasive neurochemical sensing.

## 2 Material and methods

### 2.1 Fabrication

**SCHEME 1:**
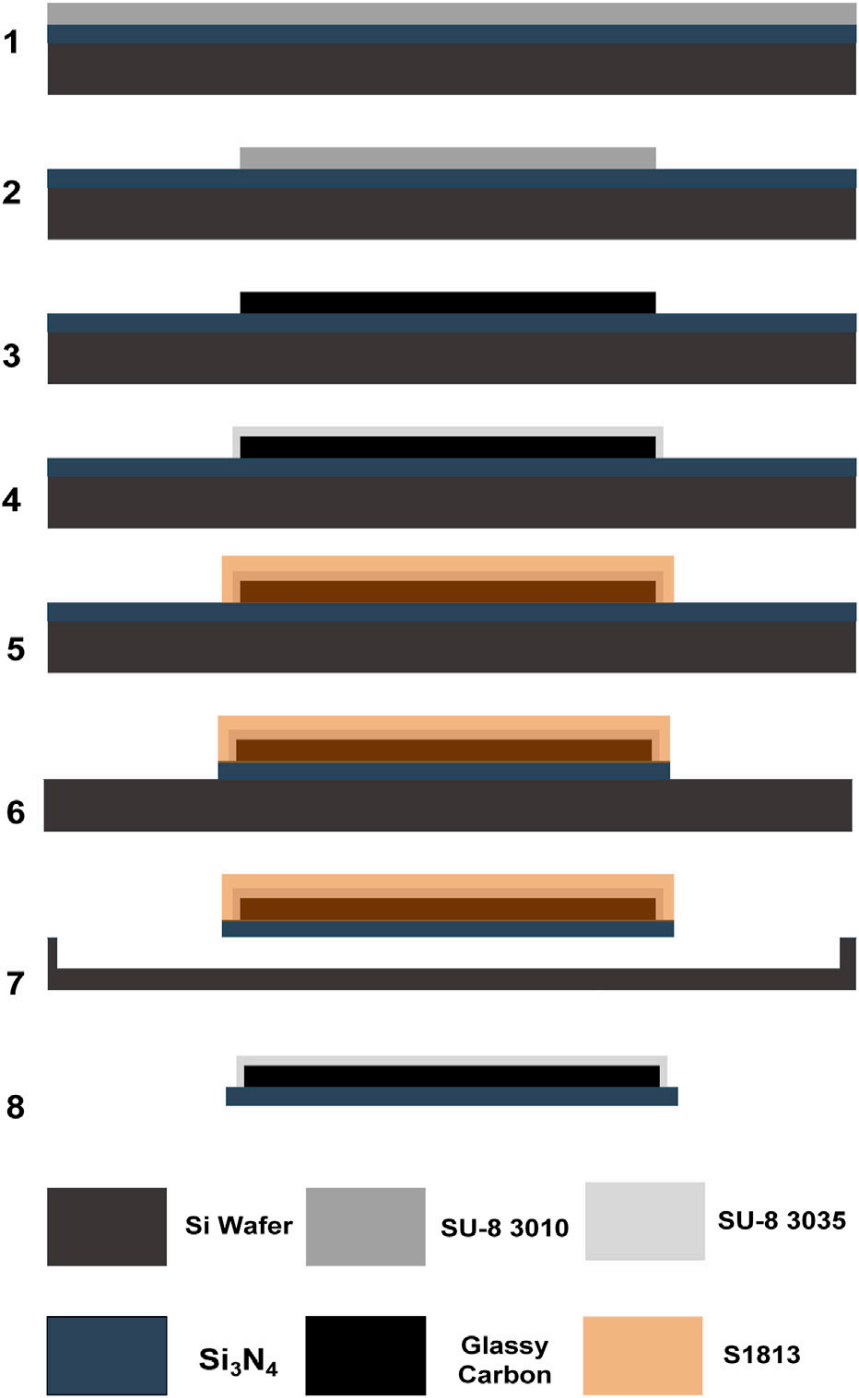
SU-8 3035 spin-coating and (2.) patterning of full glassy carbon fiber (fGCF) on Si_3_N_4_ wafer; (3.) pyrolysis; (4.) spin-coating and photolithographic patterning of the SU-8 3010 insulation layer on top of the GCF; (5.) spin-coating and photolithographic patterning of positive photoresist S1813 (protective sacrificial layer); (6.) CF_4_ reactive ion etching (RIE) of the unprotected Si_3_N_4_; (7.) pure chemical isotropic XeF_2_ etching of the exposed Si substrate to release the devices; and (8.) removal of the remaining sacrificial protective layer with acetone after the fGCF release.

The first and second steps, i.e., patterning and carbonization of SU-8 to obtain GC fibers (10 × 10 µm), are similar to previously reported (Castagnola et al., 2024; Faul et al., 2024). The fabrication process is summarized in Scheme 1. Steps 1 and 2. A 4-inch Si wafer with a 2 µm-thick low stress LPCVD Si_3_N_4_ layer (University Wafer Inc., Boston, MA, United States) was first cleaned with acetone, isopropanol, and deionized water (DI) sequentially. The wafer was then dried with an N_2_ spray gun, heated on a hot plate at 200°C for 5 min, and treated with O_2_ plasma using a reactive ion etcher (RIE, MICRO-RIE 800, Technics Inc., Anaheim, CA, United States) for 90 s at 300 mTorr pressure and 150 W power. The cleaned wafer was spin-coated with SU-8 3035 (Kayaku Advanced Materials, Westborough, MA, United States) at 2000 rpm for 1 min and soft baked at 65°C for 5 min and 95°C for 5 min. Then, the wafer was exposed using a custom-made photomask and a MA/BA6 Mask/Bond Aligner (Süss MicroTec, Garching, Germany) with a dose of 350 mJ/cm^2^.

After exposure, the wafer was first post-baked at 65°C for 3 min and 95°C for 5 min, then developed using SU-8 developer (Kayaku Advanced Materials, Westborough, MA, United States) for 1 min and cleaned with isopropanol and DI water. The patterned SU-8 was subsequently hard baked at 200°C, 180°C, and 150°C for 5 min each and allowed to cool down below 65°C. Pyrolysis of the negative SU-8 resist was performed in a high-temperature split tube furnace (STF 1200 Tube Furnace, Across International, Livingston, NJ, United States). The samples were heated to 900°C with a temperature ramp-up at a rate of 3°C/min, then maintained at 900°C under 15 standard cubic centimeters per minute (sccm) N_2_ (Airgas, Pittsburgh, PA, United States) at 0.8 Torr for 60 min. The samples were then slowly cooled to room temperature. Step 3. After the pyrolysis, the wafer was cleaned with acetone, isopropanol, and DI water sequentially and treated with O_2_ plasma with RIE for 30 s at a pressure of 300 mTorr and 120 W power. The cleaned wafer was then spin-coated with SU-8 3010 (Kayaku Advanced Materials, Westborough, MA, United States) at 3,000 rpm for 1 min and then soft based at 65°C for 3 min and 95°C for 5 min. This SU-8 layer was patterned, using a dose of 300 mJ/cm^2^, to define the insulation layer. After a post-bake at 65°C for 1 min and 95°C for 3 min, the wafer was developed using the SU-8 developer. Finally, the patterned wafer was cleaned with isopropanol and DI water, hard baked at 200°C, 180°C, and 150°C for 5 min each, and allowed to cool down below 65°C. Step 4. After the hard baking of the SU-8, the devices were protected with a sacrificial hard mask. First, the wafer was treated with O_2_ plasma with RIE for 60 s at a pressure of 300 mTorr and 100 W power and then spin-coated with a S1318 positive photoresist (MICROPOSIT™ S1800^®^ G2 Series Photoresists, Kayaku Advanced Materials, Westborough, MA, United States) at 800 rpm for 1 min and baked at 115°C for 1 min. After soft baking, the wafer was exposed and patterned with a dose of 500 mJ/cm^2^, then developed using MF-321 developer (MICROPOSIT™, Kayaku Advanced Materials, Westborough, MA, United States), cleaned with water, rinsed, and dried by N_2_ gas flow. Step 5. Then the wafer was exposed to CF_4_ reactive ion etching (RIE, 220 mTorr pressure and 200 W power) to etch the 2 µm Si_3_N_4_ layer, where not protected, leaving the Si exposed. Step 6. To release the Si_3_N_4_-insulated MEA from the Si wafer, the exposed Si was etched using a purely chemical xenon difluoride (XeF_2_) etching with pressure P(XeF_2_) of 3.5 mT at room temperature, using a xenon difluoride etching tool (Xetch X.3.B, Xactix Inc., River Park Commons, PA, United States). Step 7. After the insulated fGC fibers were released, the sacrificial protective layer was easily removed with acetone.

### 2.2 Finite element modeling

To optimize the form factor of the fGCF, we performed finite element modeling (FEM) in Autodesk Fusion 360 (San Francisco, CA, United States). A 10-mm diameter cylinder was used to model the brain tissue with Young’s modulus and Poisson’s ratio of 2 kPa and 0.45, respectively (Morin et al., 2017). The fGCF was modeled as 10 μm wide, 10 μm thick, and 3 mm long with Young’s modulus of 32.5 GPa and Poisson’s ratio of 0.17 (Garion, 2014). Adaptive meshes of second order were created and linear static stress analyses were performed to simulate the stress and displacement of the fGCF upon insertion into the brain tissue up to 3 mm.

### 2.3 Morphological characterization

Scanning electron microscope (SEM) imaging and elemental analysis of the fGCF surfaces in field-emission electron microscopy were performed using energy dispersive spectroscopy (EDS) to identify and quantify all present elements using a HITACHI S-4800 field-emission electron microscope with a Bruker (Xflash 6160) EDS attachment (HITACHI Global, Irvine, CA, United States). High-resolution optical imaging was performed using a VK-X150 3D scanning confocal microscope (Keyence America, Itasca, IL, United States). Raman spectroscopy measurements were performed using the LabRAM Soleil Raman Microscope (Horiba, Kyoto, Japan). A 532 nm laser was used with a spot size of 42.8 μm × 29.2 μm on the GC through a ×20 objective. Laser intensity was set to 25% and the scan range was 50 cm^−1^ to 3,650 cm^−1^. An average of two 15-s acquisitions was used and cosmic ray spikes were removed in LabSpec 6 software (Horiba, Kyoto, Japan).

### 2.4 Electrochemical characterization

To verify the electrode functionality, electrochemical impedance spectroscopy (EIS) and cyclic voltammetry (CV) were performed in 1 × phosphate-buffered saline (PBS, Sigma Aldrich, St. Louis, MO, United States) in a three-electrode electrochemical cell set-up with a platinum counter electrode and an Ag/AgCl wire reference electrode, using a potentiostat/galvanostat (Autolab, Metrohm, Riverview, FL, United States). EIS was performed by superimposing a sine wave (10 mV RMS amplitude) onto the open circuit potential while varying the frequency from 1 to 10^5^ Hz. During the CV tests, the working electrode potential was swept between 2 and -0.6 V vs. Ag/AgCl at a scan rate of 150 mV/s. To investigate the electron transfer kinetics of sensor surfaces, CV and EIS were conducted in the presence of 5 mM K_3_ [Fe(CN)_6_], 5 mM K_4_ [Fe(CN)_6_], and 1 M KCl as the supporting electrolyte. During the CV tests, the working electrode potential was swept between 1 V and −0.2 V vs. Ag/AgCl, with scan rates ranging from 100 mV/s to 1 V/s.

### 2.5 Fast scan cyclic voltammetry

Fast scan cyclic voltammetry (FSCV) measurements of DA were collected using a FSCV Wave Neuro potentiostat (Pine Research, Durham, NC, United States) and analyzed using HDCV software (University of North Carolina at Chapel Hill, Chapel Hill, NC, United States). The electrode was scanned using a triangular waveform (−0.4 to 1.3 to −0.4 V vs. Ag/AgCl) at 10 Hz and 400 V/s scan rate. *In vitro* DA (Dopamine hydrochloride, >98.0%, Sigma-Aldrich, St. Louis, United States) calibration were performed using freshly prepared DA standard solutions dissolved in 1× PBS. Electrodes were calibrated using 0.25 μM–2 μM DA concentrations. The different concentrations were diluted starting from a freshly prepared 1 mM DA solution. DA detection was identified by inspection of background-subtracted cyclic voltammograms. Electrode sensitivities were determined by the linear regression slope of the maximum oxidation current vs. DA concentration calibration plots.

### 2.6 Square wave voltammetry

Electrochemical detection of DA was performed via SWV, similarly to our previous study (Taylor et al., 2019; Castagnola et al., 2022; Castagnola et al., 2024). SWV experiments were carried out using a potentiostat/galvanostat (AutoLab, Metrohm, Utrecht, Netherlands) connected to a three-electrode electrochemical cell with a platinum counter electrode and an Ag/AgCl reference electrode. The SWV waveform was repeatedly applied from −0.2 V to 0.3 V with a 25 Hz step frequency, a 50 mV pulse amplitude, and a 5 mV step height every 15 s. The potential was held at 0 V between scans. *In vitro* DA calibrations were performed using freshly prepared DA solutions dissolved in 1 × PBS in a 50 nM−1 μM concentration range. Electrode sensitivity was determined by the slope of the linear range of the calibration plot relating the DA peak current at 0.15 V to the DA concentration.

### 2.7 *In vivo* experiments

*In vivo* performance was determined through acute experiments conducted in the dorsal striatum (DS) of mice (C57BL/6J, 8–12 weeks, 30–35 g; Jackson Laboratory, Bar Harbor, ME, United States). All animal care and procedures were performed under approval of the Louisiana Tech University Institutional Animal Care and Use Committee and in accordance with regulations specified by the Division of Laboratory Animal Resources. Mice were induced with 1.5%–2% isoflurane mixed with oxygen flow at 1 L/min, then maintained at 1.25%–1.5%. Body temperature was maintained at 37°C with a thermostatically controlled heating pad (Harvard Apparatus, Holliston, MA, United States).

After the animal head was fixed in a stereotaxic frame (Narishige International United States, Inc. Amityville, United States), the skin and connective tissue on the surface of the skull were removed. A small pinhole craniotomy was made over the DS (1 mm anterior to bregma, and 1.5 mm lateral from midline) with a high-speed dental drill (0.007 drill bit, Fine Science Tools, Inc., Foster City, CA, United States), and bone fragments were carefully removed with forceps and saline. Saline was applied continuously onto the skull to dissipate heat from the high-speed drill. This procedure is similar to what previously reported (Taylor et al., 2019; Castagnola et al., 2022).

For SWV measurements, GCFs were lowered 3.0 mm below the cortical surface into the DS using a micromanipulator. Two additional small pinhole craniotomies were performed for the introduction of the Ag/AgCl reference electrode contralaterally to the GCF and a bone screw counter electrode caudally to the reference. EIS was measured immediately after the MEA implantation. Then, the tonic DA response was measured using the SWV waveform over a 40 min period. To confirm the chemical specificity of our measurements, following 10 min of data collection, mice were administered with 2 mg/kg intraperitoneal (i.p.) raclopride, a selective antagonist on D2 dopamine receptors (Sigma Aldrich, St. Louis, MO, United States), and 20 mg/kg i.p. nomifensine, a dopamine reuptake inhibitor (Sigma Aldrich, St. Louis, MO, United States) (Castagnola et al., 2022; Walters et al., 2016). Upon reaching the predetermined experimental endpoint, the MEAs were explanted, and the animals were humanely sacrificed using approved procedures.

SWV and EIS experiments were acquired using a potentiostat/galvanostat (Autolab PGSTAT128N, Metrohm, Utrecht, Netherlands) connected to the three-electrode configuration: working electrode, bone screw (counter electrode), and Ag/AgCl wire reference electrode. DA peaks were isolated from the nonfaradaic background current for each SWV scan by subtracting a modeled polynomial baseline, using a previously described methodology (Taylor et al., 2019). DA concentration was determined for all *in vivo* experiments by converting the SWV peak current to the DA concentration using the pre-calibration electrode sensitivity, as previously reported (Taylor et al., 2019; Castagnola et al., 2022).

Proof-of-principle *in vivo* experiments were performed to evaluate the FSCV performance of the fGCFs. The fGCFs were lowered 3.0 mm below the cortical surface into the DS using a hand-driven micromanipulator. An additional small pinhole craniotomy was performed for the introduction of the Ag/AgCl reference electrode contralaterally to the fGCF. A second portion of skull and dura was removed for the introduction of a bipolar stainless-steel stimulating electrode (MS303/a; Plastics One, Roanoke, VA, United States), positioned over the medial forebrain bundle (MFB; 1.6 mm posterior to bregma, 1 mm lateral from bregma, and 4.8 mm below cortical surface). MFB stimulation was conducted via the application of an optically isolated stimulus waveform (Neurolog 800, Digitimer, Letchworth Garden City, United Kingdom) consisting of a biphasic, constant-current square wave (2 ms per pulse, 250 µA pulse height, 10 Hz frequency, 15 pulses) with the bipolar stainless-steel electrode. Fast scan cyclic voltammetry (FSCV) was performed with a 4-channel Wave Neuro potentiostat (Pine Research, Durham, NC, United States), and the data were collected and analyzed using HDCV software (University of North Carolina at Chapel Hill, NC, United States). The electrode was scanned using a triangular waveform with a negative holding potential of −0.4 V, a 1.3 V switching potential, and applied using a 400 V/s scan rate at 10 Hz.

For assessment of the device tissue interface, we implanted fGCFs in both hemispheres of a male Sprague-Dawley rat (250–350 g, Charles River, Wilmington, MA). The rat was anesthetized with isoflurane (5% for induction, 2.5% for maintenance), placed on a thermal pad, and the head was fixed into a stereotaxic frame (Kopf Instruments, Tujunga, CA, United States). Standard aseptic surgical procedures were used as approved by the Institutional Animal Care and Use Committee of the University of Pittsburgh. A midline incision was made in the scalp and connective tissue was removed, and burr holes were drilled in the skull between bregma and lambda. Additional burr holes were made for bone screws to anchor the head cap afterward. The fGCFs were then implanted and secured with Kwik-Sil (World Precision Instruments, Sarasota, FL) and blue-light curing dental cement. Once the head cap was fully secured, the scalp was sutured back together. After 1 week, the rat was transcardially perfused with PBS, followed by 4% paraformaldehyde. The brain was then extracted and dehydrated in 15% and 30% sucrose, sequentially. The removed brain tissue was cryoprotected using optimal cutting temperature compound (OCT, Fisher Healthcare, Houston, TX), frozen, and sectioned. Tissue sections were hydrated in PBS and stained for neuronal cell body (1:500 mouse anti-NeuN, Millipore, Billerica, MA), apoptotic cell death rabbit (1:500 Asp175, Cell Signaling Technology, Boston, MA), and blood-brain barrier injury (1:500 goat anti-rabbit IgG, Invitrogen, Carlsbad CA). DAPI was used as counter stain for cell nuclei. The tissue sections were imaged using a Confocal Laser Scanning Microscope Fluoview FV3000 at the Center for Biologic Imaging at the University of Pittsburgh.

## 3 Results and discussion

**FIGURE 1:**
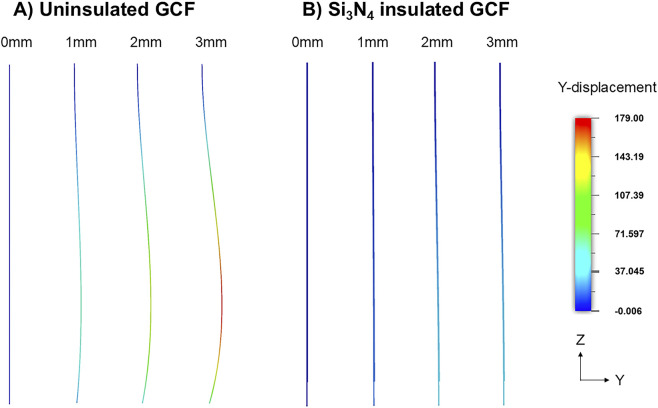
Finite element simulation demonstrating **(A)** buckling of the uninsulated GCF and **(B)** straight insertion of silicon nitride insulated GCF at 1 mm, 2 mm, and 3 mm insertion depths. The color scale shows the lateral displacement in Y direction of the ZY view of the snapshots.

FEM was used to optimize the form factor for aid-free implantation of fGCFs into the mouse DS. The model illustrated that standalone fGCF cannot be inserted deep into the brain tissue without buckling (Figure 1A). Significant buckling with ∼179 μm maximum lateral displacement was observed upon simulated insertion of 3 mm of standalone uninsulated fGCF, whereas fGCF insulated with 2 μm thick Si_3_N_4_ and 10 μm thick SU-8 was able to self-support and withstand buckling (Figure 1). Minimal displacement (≤37 μm) of the insulated fGCF allows for precise targeting of the striatum with little insertion damage. From this, we determined the form factor of the fGCFs for *in vivo* application in the dorsal striatum.

A double-etching microfabrication technique, recently developed by our lab (Faul et al., 2024), was employed to batch-fabricate fGCFs, as shown in Scheme 1. The process began with patterning the fGCF on a Si₃N₄-coated wafer using photolithography. Following patterning, the fibers were carbonized at 900°C in a tube furnace with a controlled environment, and leaving the carbon tip exposed for sensing, rest of the electrode was insulated with SU-8 to prevent undesired electrical contact. A sacrificial layer was used to protect both the insulation and the GC microstructures during subsequent etching steps. Two distinct gases were used for the selective etching of the Si₃N₄ and silicon wafer. Tetrafluoromethane (CF₄) selectively etched the exposed Si₃N₄, while xenon difluoride (XeF₂) was employed to selectively etch the underlying silicon wafer. The process took advantage of the 200:1 Si vs. Si₃N₄ etch selectivity (Faul et al., 2024; Winters and Coburn, 1979; Arana et al., 2007), ensuring that the Si₃N₄ layer remained intact as an insulation barrier throughout the fabrication. We observed that during the pyrolysis, the hard-baked SU-8 precursor experienced about 70% height shrinkage, similar to what we previously observed (Faul et al., 2024). This factor should be considered for the choice of SU-8 viscosity and spinning rate during the photolithography. Controlling these parameters effectively is crucial for achieving the desired thickness and uniformity of the SU-8 layer, which ultimately determines the dimensions and properties of the fabricated fGCFs.

**FIGURE 2:**
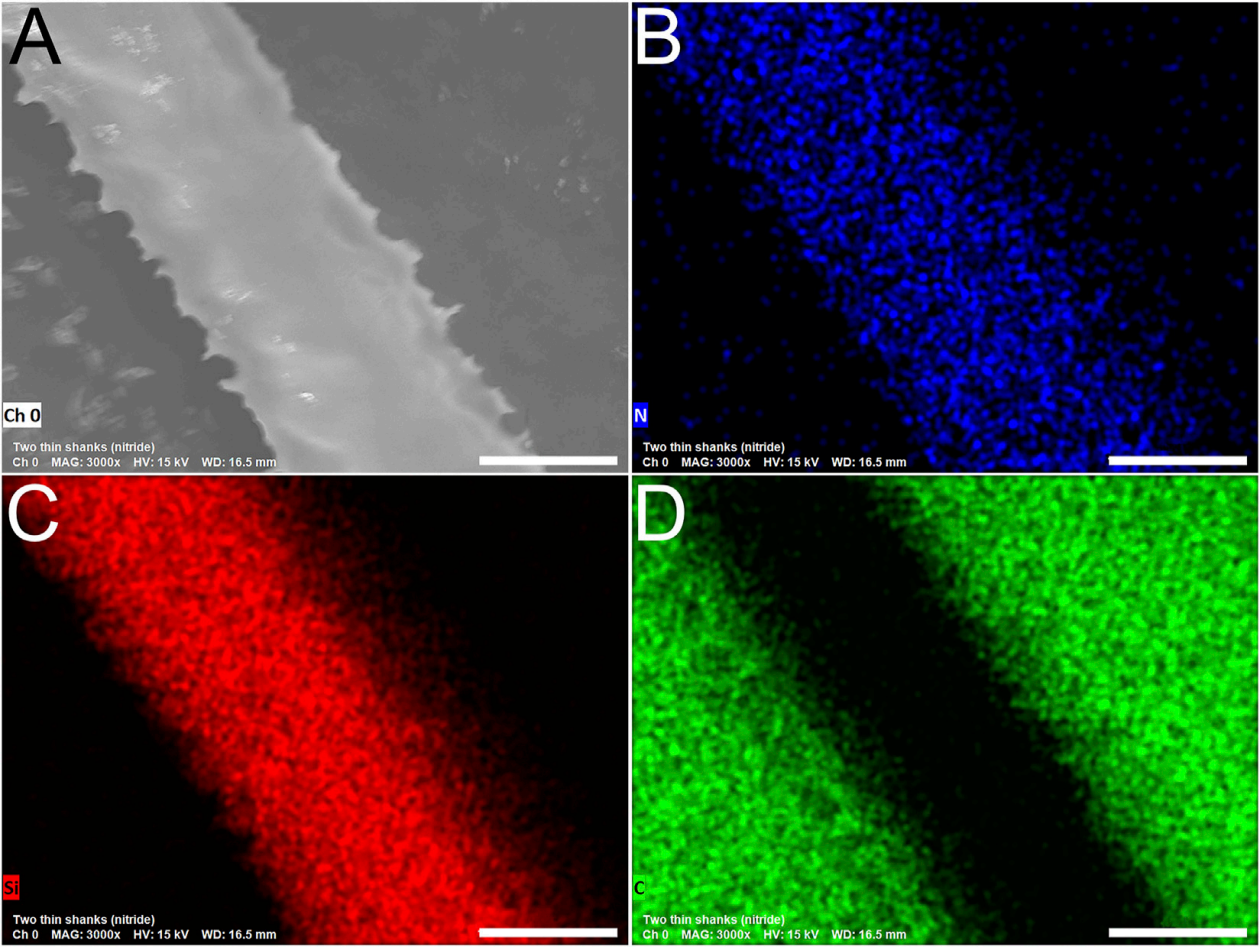
SEM image **(A)** and elemental analysis **(B–D)** of the surface of fGCF release using isotropic XeF_2_ etching (side insulated with Si_3_N_4_). Elemental analyses conducted using energy dispersive X-ray spectroscopy (EDS) confirmed a consistent and uniform presence of N [in blue, **(B)**] and Si [in red, **(C)**] along the fGCF and the presence of carbon in the areas where the focus of the analysis was on the carbon tape of the stub [green, **(D)**], where the fGCF is positioned for imaging. Scale bar is 9 µm.

To confirm the presence and uniformity of the Si₃N₄ insulation layer, we used scanning electron microscopy (SEM) and energy-dispersive X-ray spectroscopy (EDS). EDS elemental mapping revealed the presence of silicon (red, Figure 2C), and nitrogen (blue, Figure 2B) uniformly distributed across the insulated regions of the fGCF, consistent with the expected composition of the silicon nitride layer. In Figure 2D, when the analysis focused on the carbon tape attached to the SEM stub not covered by the fGCF, a strong carbon signal (green) was detected while the insulated fGCF did not show carbon signal confirming the selectivity of the XeF₂ etching process and the integrity of the insulated device. Quantitative EDS analysis with a relative percentage of chemical elements is reported in Supplementary Table S1.

**FIGURE 3:**
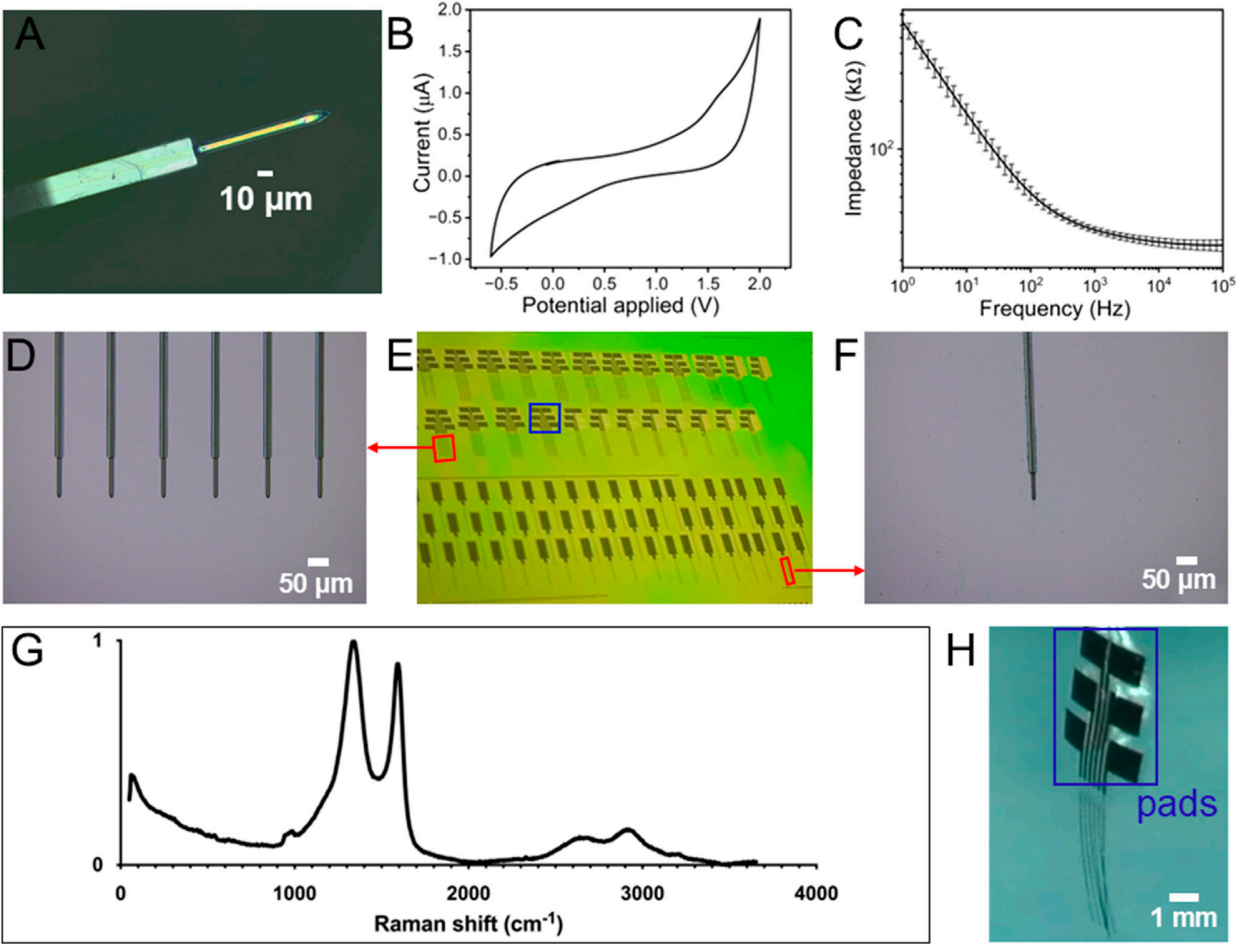
**(A)** Optical picture of fGCF (10 µm wide, 10 µm thick); **(B)** Representative example of a cyclic voltammetry plot of fGCF; **(C)** Electrochemical impedance spectra of the magnitude impedance of fGCFs (mean and SD, n = 5); **(D)** Optical picture of fGCF array with six GCFs at 170 μm each; **(E)** Batch fabricated fGCF and fGCF arrays in a Si_3_N_4_ coated 4 in wafer before being released; **(F)** Optical picture of fGCF in a Si_3_N_4_ coated wafer; **(G)** Raman Spectrum of GC; **(H)** Optical picture of a released fGCF array.

The resulting fGCFs exhibited miniaturized features with 10 μm in width, 10 μm in thickness and 150 μm length of the GC standing out from the SU-8 insulator, as shown in Figure 3A. The fGCFs were batch-fabricated on a 4-inch silicon wafer as shown in Figure 3E, with up to 80 devices per batch including single fGCF and fGCF arrays, highlighting the scalability and reproducibility of the GC fine structures. Arrays of fGCFs were fabricated with four to six fGCFs at 170 μm each as shown in Figure 3D and H which can be used to detect signals from multiple locations simultaneously. The total length of each fGCF shank is 3 mm.

Electrochemical characterization of the fGCFs was performed using electrochemical impedance spectroscopy (EIS) and cyclic voltammetry (CV) to assess electrode properties, insulation quality, and fabrication consistency. EIS measurements (Figure 3C), performed in the 1Hz-100 kHz range, presents impedance values of 30.30 ± 1.38 kΩ at 1 kHz and 24.88 ± 2.05 kΩ at 100 kHz, confirming good electrode conductivity. The Nyquist plots shown in Supplementary Figure S1 are in agreement with the results reported in the literature for capacitive microelectrodes (Nimbalkar et al., 2018; Vomero et al., 2017). The cyclic voltammogram of fGCFs in 1 × PBS (Figure 3B) exhibits an approximately rectangular current response, predominantly governed by double-layer capacitance, and demonstrates a broad electrochemical window extending up to 1.7 V without triggering hydrolysis reactions, a characteristic feature of carbon electrodes with capacitive behavior (Faul et al., 2024; Nimbalkar et al., 2018; Castagnola et al., 2021b). This wide potential range ensures the electrode’s suitability for FSCV, enabling precise neurotransmitter detection without compromising background stability. Additionally, characterization measurements were performed by CV and EIS in the presence of 5 mM K_3_ [Fe(CN)_6_], 5 mM K_4_ [Fe(CN)_6_], a well-known redox couple used to investigate the electron transfer kinetics of sensor surfaces (Liv, 2024b; Liv and Demirel, 2024; Liv et al., 2023; Liv and Karakus, 2023; Billa et al., 2024). The CV plots display typical redox peaks at 0.2 and 0.06 V vs. Ag/AgCl, respectively (Supplementary Figure S2A). The absence of a semicircle at high frequencies in the Nyquist plots indicates a low charge transfer resistance (Rct), suggesting that fGCFs promote fast and efficient electron transfer between the electrode and the surrounding redox species, which enhances their accurate detection capability (Supplementary Figure S2B).

Next, Raman spectroscopy was used to identify the structural fingerprint of the GC (Figure 3G). The Raman spectrum shows two primary peaks typical for carbon materials: the D band at 1,323 cm^−1^ and the G band at 1,604 cm^−1^. The D band, associated with the breathing mode of six-atom rings, indicates defects such as edge-plane boundaries or doping that disrupt the graphene structure. The G band is the E_2g_ phonon mode, reflecting sp^2^ graphitic carbon structures. From the Raman spectrum, the defect level or the D/G peak height ratio of the GC pyrolyzed from SU-8 3,035 was calculated to be ∼1.1, which is comparable to GC obtained from other varieties of SU-8 and pyrolyzed using similar conditions (Castagnola et al., 2024; Castagnola et al., 2023). The Raman spectra also show some secondary peaks: the 2D peak, which is the overtone of the D peak, is at 2,642 cm^−1^, and the D + D′ peak at 2,916 cm^−1^ (Ferrari and Basko, 2013), indicating high ordering and graphitization in the pyrolyzed GC (Tyler et al. 2023). The defects and the high graphitization level promote adsorption and electron transfer in carbon materials (Cao et al., 2019). Following Raman and electrochemical characterization, the ability of the fGCF to detect phasic DA concentrations was evaluated *in vitro* using FSCV. fGCF were scanned using a triangular waveform commonly used for DA detection, with a negative holding potential of −0.4 V, a 1.3 V switching potential, and back to the holding potential to oxidize DA and reduce DAoQ, applied using a 400 V/s scan rate at 10 Hz (Venton and Cao, 2020; Castagnola et al., 2022; Bath et al., 2000; Robbins et al., 2022). The holding potential of −0.4 V is commonly applied to the working electrode to selectively preconcentrate cationic DA on the electrode surface (Puthongkham and Venton, 2020; Venton and Cao, 2020), and the switching potential of 1.3 V has shown to increase the DA sensitivity, activating the carbon surface without generating electrolysis of water (Puthongkham and Venton, 2020; Heien et al., 2003; Takmakov et al., 2010).

**FIGURE 4:**
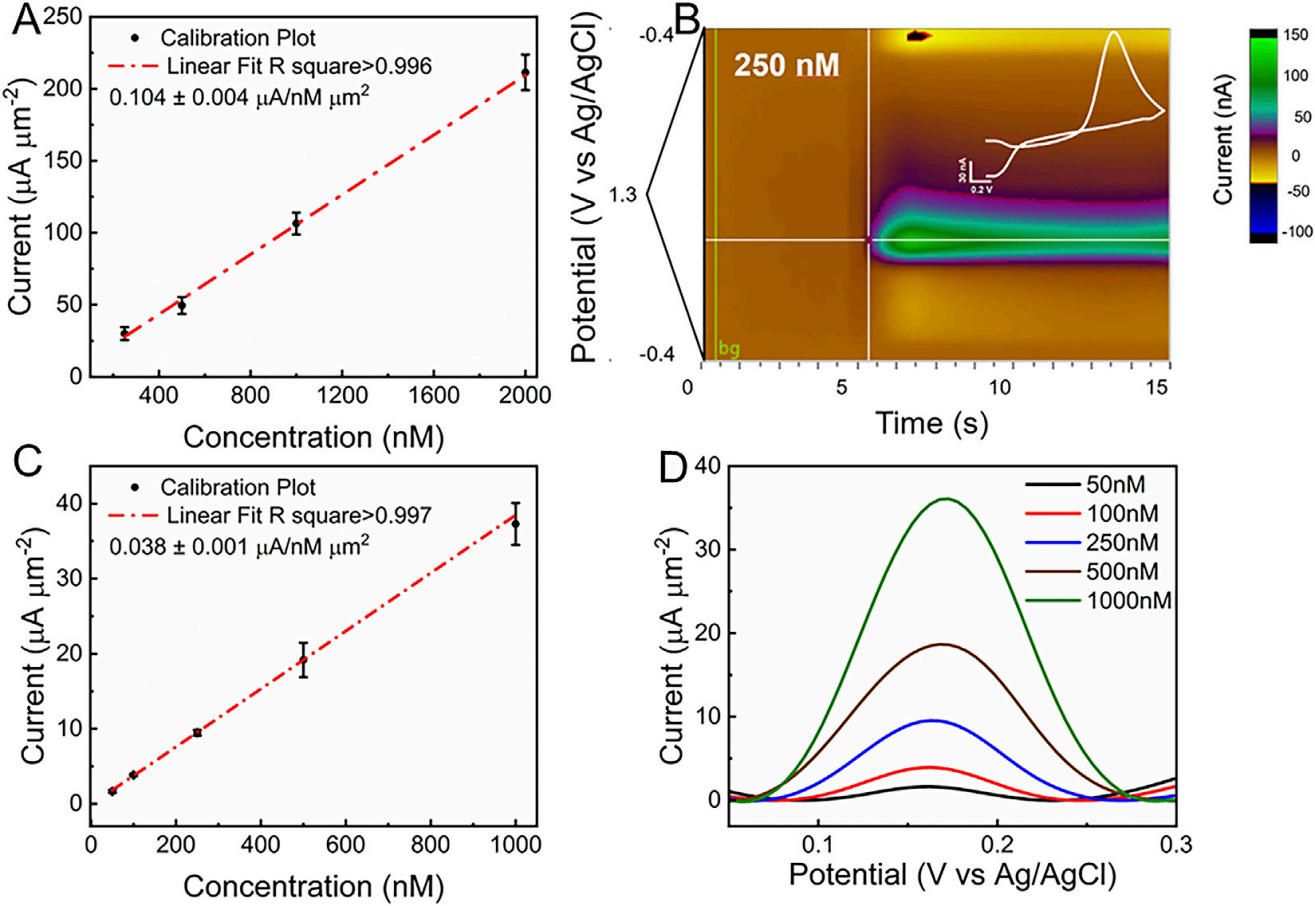
**(A)** *In vitro* FSCV calibration plot of fGCFs conducted in 1xPBS in the DA concentration range of 0.25 μM - 2 μM (peak current vs. DA concentration, mean and SD; n = 5), **(B)** Color plot and background subtracted FSCV plot (inset, in white) corresponding to 250 nM DA concentration; **(C)** In vitro SWV DA calibration plot (Peak current vs. DA concentration, mean and SD, n = 5) obtained from fGCF conducted in 1x PBS in 50 nM -1,000 nM concentration range and **(D)** corresponding baseline subtracted SWV DA peaks. Representative SWV without baseline subtraction are reported in Supplementary Figure S3.

Different DA concentrations, ranging from 0.25 μM to 2 μM, were tested according to the expected physiological range for phasic release (Castagnola et al., 2022; Mitch Taylor et al., 2012; Robinson et al., 2014; Wu et al., 2001). Figure 4A shows the calibration plot for fGCFs conducted in 1x PBS within the 0.25 μM–2 μM DA concentration range. The fGCF exhibited high sensitivity to DA (0.104 ± 0.004 μA/nM μm^2^), with linear calibration curve (r^2^ > 0.99). Because FSCV generates a large volume of data, performing one CV cycle every 100 milliseconds, a common approach to visualizing this data is through a color plot, which condenses the information into a two-dimensional representation. The X-axis represents time, the Y-axis represents the applied voltage, and the pseudo-colors indicate the current magnitude at each point, with different color and color intensities to current levels associated with DA oxidation and reduction. Figure 4B shows a representative color plot generated with 250 nM DA concentration, where pseudo-colors depict the oxidation and reduction of DA. The inset plot in Figure 4B presents the background subtracted CV, highlighting the characteristic reduction (−0.17 V) and oxidation (0.63 V) DA peaks. We previously demonstrated the FSCV detection capability of planar GC and GCF MEAs (Castagnola et al., 2022; Castagnola et al., 2024; Castagnola et al., 2021a; Nimbalkar et al., 2018). GC presented high sensitivity toward DA, attributed to the presence of curved graphene-like layers and dense edge planes formed during SU-8 pyrolysis at >900°C, rich in functional groups, which have also been shown to increase hydrophilicity and reduce fouling (Castagnola et al., 2024; Castagnola et al., 2021a). The theoretical lower detection limit (LOD), defined as three times the standard deviation of the noise (Taylor et al., 2019; Castagnola et al., 2021a), was estimated to be 3.45 ± 0.36 nM for DA using full GCF when using FSCV, similar to previously reported values for GCF-MEAs and GC-MEAs (Castagnola et al., 2024; Castagnola et al., 2021a).

To assess whether the fGCF could detect tonic DA levels, SWV measurements were carried out *in vitro* using the previously optimized square waveform for DA (Taylor et al., 2019; Castagnola et al., 2022; Castagnola et al., 2024). DA concentrations ranging from 50 nM to 1 μM were tested to encompass the expected DA physiological range (Taylor et al., 2019; Gonon et al., 1984; Atcherley et al., 2013; Atcherley et al., 2015; Johnson et al., 2018; Castagnola et al., 2022; Wu et al., 2023). A clear DA peak at approximately 0.16 V was observed in each concentration, with amplitude increasing proportionally with concentration, as shown in Figure 4D. To isolate DA peaks from the non-faradaic background current, a modeled polynomial baseline was subtracted from each SWV scan using a previously established method (Taylor et al., 2019). The calibration plot is shown in Figure 4C relates the DA peak current at 0.16 V (mean and standard deviation, n = 5) to DA concentration, demonstrating linear DA detection within the 10 nM to 500 nM range (r^2^ > 0.99). High DA sensitivity of 0.038 ± 0.001 μA/nM μm^2^ was achieved, determined from the slope of the linear region of the calibration plot. The theoretical lower detection limit (LOD), defined as three times the standard deviation of the noise (Taylor et al., 2019; Castagnola et al., 2021a), was estimated to be 7.65 ± 0.55 nM for DA using full GCF when applying SWV, similar to what previously reported using PEDOT/CNT functionalized CFEs (Taylor et al., 2019), which is far below tonic DA concentrations reported *in vivo* (Taylor et al., 2019; Atcherley et al., 2013; Atcherley et al., 2015; Oh et al., 2016; Oh et al., 2018; Castagnola et al., 2022; Castagnola et al., 2024).

**FIGURE 5:**
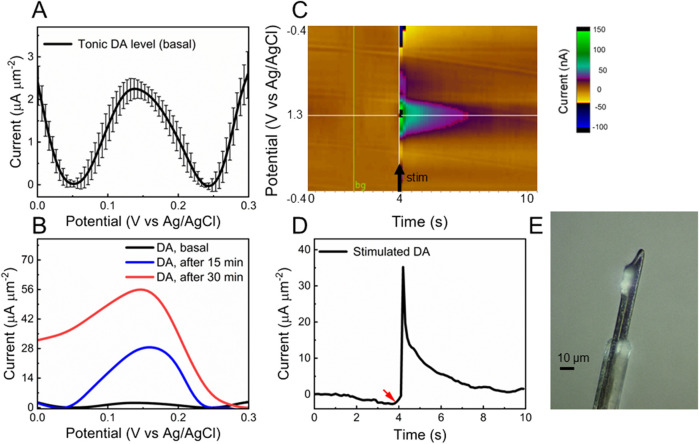
*In vivo* validation. **(A, B)** In vivo baseline subtracted SWV of **(A)** tonic DA peak before (average and standard deviation, n = 3 mice; the recording, for each mouse, corresponds to SWV measurements collected over 20-min recordings) and **(B)** 15 min (blue) and 30 min (red) post-administration of a cocktail of 2 mg/kg raclopride (RAC) and 20 mg/kg nomifensine (NOM). **(C)** In vivo FSCV color plot corresponding to DA release evoked by electrical stimulation of DA axons in the MFB; and **(D)** corresponding time/current plot. Red arrow shows when the stimulation started. **(E)** Optical picture of fGCF explanted from the brain.

Before proceeding with *in vivo* DA detection, the insertion capability of the fGCF was evaluated to confirm its ability to self-penetrate tissue without the need for tungsten wires. The fGCF was inserted into the DS of isoflurane-anesthetized mice, successfully self-penetrating along a straight trajectory without assistance, as predicted by the finite element model (see Supplementary Video). The electrode was explanted without any observed structural damage, confirming the robustness of the standalone fGCF design to sustain penetration. A representative optical picture of a fGCF immediately after brain extraction is reported in Figure 5E.

The *in vivo* capability of fGCFs for tonic DA detection was determined through acute surgical experiments conducted in the DS of isoflurane-anesthetized mice. The fGCF was implanted at a 3 mm depth in the DS, and the tonic DA response was measured using the SWV waveform described earlier. Tonic DA levels were continuously monitored for 20 min to establish a baseline. Then, to validate chemical specificity, mice were intraperitoneally (i.p.) administered with a cocktail of 2 mg/kg raclopride (RAC) and 20 mg/kg nomifensine (NOM), both known to selectively increase extracellular DA levels. Figure 5A shows the baseline DA peak recorded from the DS (average and standard deviation over 20-min recordings, n = 3). The DA signal increased progressively after drug administration. The blue curve in Figure 5B represents the DA peak 15 min post-administration, while the red curve indicates the maximum response peak after approximately 30 min, confirming the fGCF’s capability to detect tonic DA and changes in tonic DA levels in real time. Converting the current values into concentrations using the pre-calibration curve, similar to a previous reported by our group (Taylor et al., 2019; Castagnola et al., 2022; Castagnola et al., 2024), we estimate a DA basal level of 60.90 ± 4.36 nM in the DS, that increases up to 323.23 ± 12.6 nM after 15 min from drug administration and 636.26 ± 22.6 nM after 30 min. The tonic DA concentration in the DS measured with fGCFs is comparable to levels previously obtained using the same SWV techniques at PEDOT/CNT coated GC MEAs (56.2 ± 12.3 nM) (Castagnola et al., 2022) and uncoated GCFs (40.11 ± 3.35 nM) (Castagnola et al., 2024) in the mouse DS. It is slightly lower, but comparable, to DA concentrations measured via SWV at PEDOT/CNT-coated CFEs in the rat DS (82 ± 6 nM) (Taylor et al., 2019). These measurements are consistent with FSCAV (90 ± 9 nM mouse nucleus accumbens) (Atcherley et al., 2015), convolution-based FSCV (41 ± 13 nM, rat nucleus accumbens) (Johnson et al., 2017), and CBM-FSCV (73 ± 5 nM, rat striatum) (Rusheen et al., 2020; Oh et al., 2016).

We also evaluated *vivo* detection of phasic DA release using FSCV. A stimulating electrode was lowered into the MFB of an isoflurane-anesthetized mouse to stimulate nigrostriatal dopaminergic neurons, while a fGCF was implanted into the DS to detect the consequent DA overflow, as described in detail in the materials and methods section. An example of FSCV detection obtained using a fGCF in the DS is reported in Figures 5C, D. Figure 5C shows a color plot of the DA released in the DS, evoked by electrical stimulation of axons in the MFB, without pharmacological alteration. Figure 5D reports the corresponding current ∼ time plots, showing the stimulation-evoked increase in the detected background-subtracted FSCV signal, with a subsequent return to baseline over several seconds upon the cessation of stimulation. Converting the current values into concentrations using the pre-calibration curve (Castagnola et al., 2022), we obtained an average concentration of 357.14 nM, in line with the values previously reported in the literature for phasic DA release evoked by MFB stimulations, without pharmacological manipulation (Wu et al., 2001).

**FIGURE 6:**
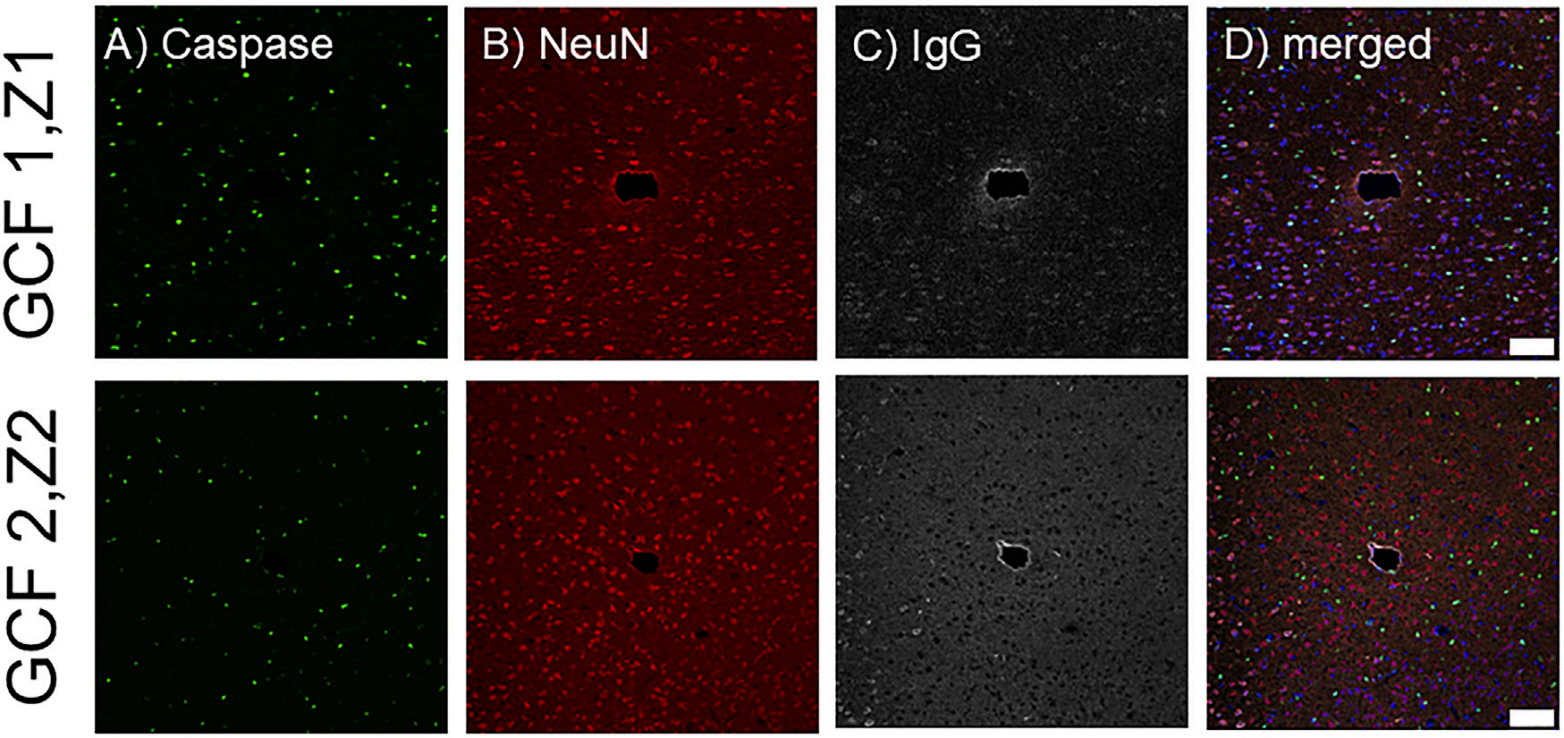
Histological analysis of the tissue surrounding the fGCF after 1 week. Images show the electrode tracks from two different implants (GCF1 and GCF2) at a depth of Z1 = -1,225 and Z2 = -1,325 µm, respectively stained for **(A)** Caspase (green), **(B)** NeuN (red), **(C)** IgG (gray), and **(D)** merged including DAPI in blue. Scale bar is 100 µm.

Next, we assessed the tissue response to implanted fGCFs using immunohistochemistry at 1 week (Figure 6). Qualitatively, we found an increased IgG intensity immediately surrounding the fGCF implants (Figure 6C). IgG (immunoglobulin G) is protein normally present in the vasculature and can leak into the brain where blood-brain barrier (BBB) is compromised. IgG staining, therefore, has been used as a marker for BBB leakage and has been commonly observed near neural implants (Du et al., 2017). We observed a thin IgG staining zone immediately near the probe indicating minimum insertion injury by the fGCF implant. Furthermore, unlike the lowered neuronal density and increased number of apoptotic cells commonly found near microelectrode implants, we observed no difference in neuron density or caspase positive cells near the implant vs. distant from the implant at different depths (Figures 6A, B). This indicates minimum tissue damage due to insertion and good electrode-tissue integration, which is supported by the ability to detect DA release from neurons surrounding the fGCFs using FSCV, without the need of pharmacological manipulation, as FSCV measurements are known to be extremely sensitive to tissue damage (Roberts and Sombers, 2018; Jaquins-Gerstl and Michael, 2015).

## 4 Conclusion

In this study, we introduced a double dry etching technique to fabricate fGCF and fGCF arrays for tonic and phasic DA detection with minimal tissue damage. This method enables the batch fabrication of GC fibers and interconnects from a single homogeneous material, eliminating the need for metal interconnections and addressing possible concerns about electrical and mechanical stability under prolonged electrochemical cycling. Finite element modeling was used to optimize the form factor, enabling aid-free implantation of fGCFs in the mouse DS. fGCFs present subcellular size, biocompatibility, and exceptional electrochemical properties, combined with the reproducibility, versatility, and scalability of photolithography-based batch production. They demonstrated real-time detection of tonic DA levels using SWV, as well as electrically evoked DA release using FSCV *in vivo*. This work offers a promising solution for high-performance, minimally invasive neurochemical sensing.

## Data Availability

The raw data supporting the conclusions of this article will be made available by the authors, without undue reservation.
