# Supplementary material for: Batch-fabricated full glassy carbon fibers for real-time tonic and phasic dopamine detection
Source: Front Bioeng Biotechnol. 2025 Feb 28;13:1543882. doi: 10.3389/fbioe.2025.1543882 (PMC11906454; doi:10.3389/fbioe.2025.1543882)
Supplement: Supplementary file 1 [file Table1.docx]

Supplementary Information

**Batch-fabricated Full Glassy Carbon Fibers for Real-time Tonic and Phasic Dopamine Detection**

Umisha Siwakoti^1^, May Yoon Pwint^2,3^, Austin M. Broussard^1^, Daniel R. Rivera^1^, X. Tracy Cui^2,3,4^, Elisa Castagnola^1,2,5^

^1^ Department of Biomedical Engineering, Louisiana Tech University, 818 Nelson Ave, Ruston, LA, 71272, USA

^2^ Department of Bioengineering, University of Pittsburgh, 3501 Fifth Ave., Pittsburgh, PA, 15260, USA

^3^ Center for Neural Basis of Cognition, University of Pittsburgh, 4400 Fifth Ave, PA 15213, Pittsburgh, PA, 15261, USA

^4^ McGowan Institute for Regenerative Medicine, University of Pittsburgh, 450 Technology Drive, Pittsburgh, PA, 15219-3110, USA

^5^ Institute for Micromanufacturing, Louisiana Tech University, 911 Hergot Ave, Ruston, LA 71272, USA

*** Correspondence:** elisa@latech.edu

Keywords: glassy carbon fibers, microelectrodes, dopamine, fast scan cyclic voltammetry (FSCV), square wave voltammetry (SWV).

| **Element** | **Mass Normalized (%)** | **Abs Error (%)** | **Rel. Error (%)** |
| --- | --- | --- | --- |
| Carbon | 55.26±2.35 | 9.43±0.27 | 11.53± 0.19 |
| Nitrogen | 16.66± 0.28 | 3.71±0.13 | 15.06 ±1.03 |
| Oxygen | 9.22±0.39 | 1.97±0.01 | 14.42±0.73 |
| Silicon | 18.85±2.46 | 1.20±0.25 | 4.26±0.03 |

**Supplementary Table 1**. EDS analysis with a relative percentage of chemical elements.


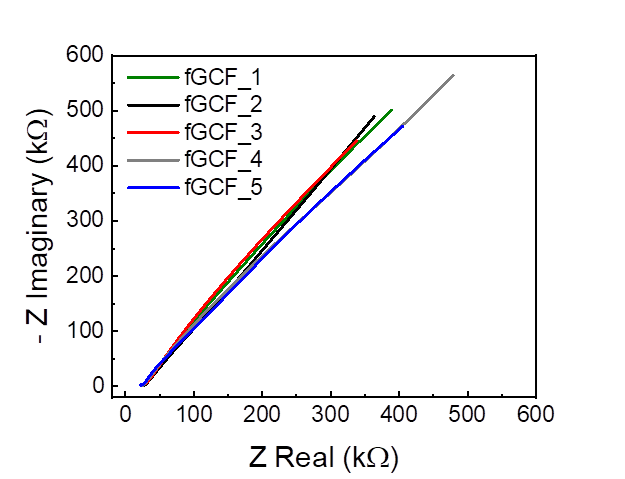


**Supplementary Figure 1.** Nyquist plots of full glassy carbon fibers (fGCFs) in 1xPBS. These plots correspond to the impedance data presented in Figure 3C of the main text. The absence of a semicircle at high frequencies in the Nyquist plots indicates a low charge transfer resistance (Rct), indicating minimal or absent faradaic processes. This observation is consistent with previously reported data for purely capacitive electrodes (1).





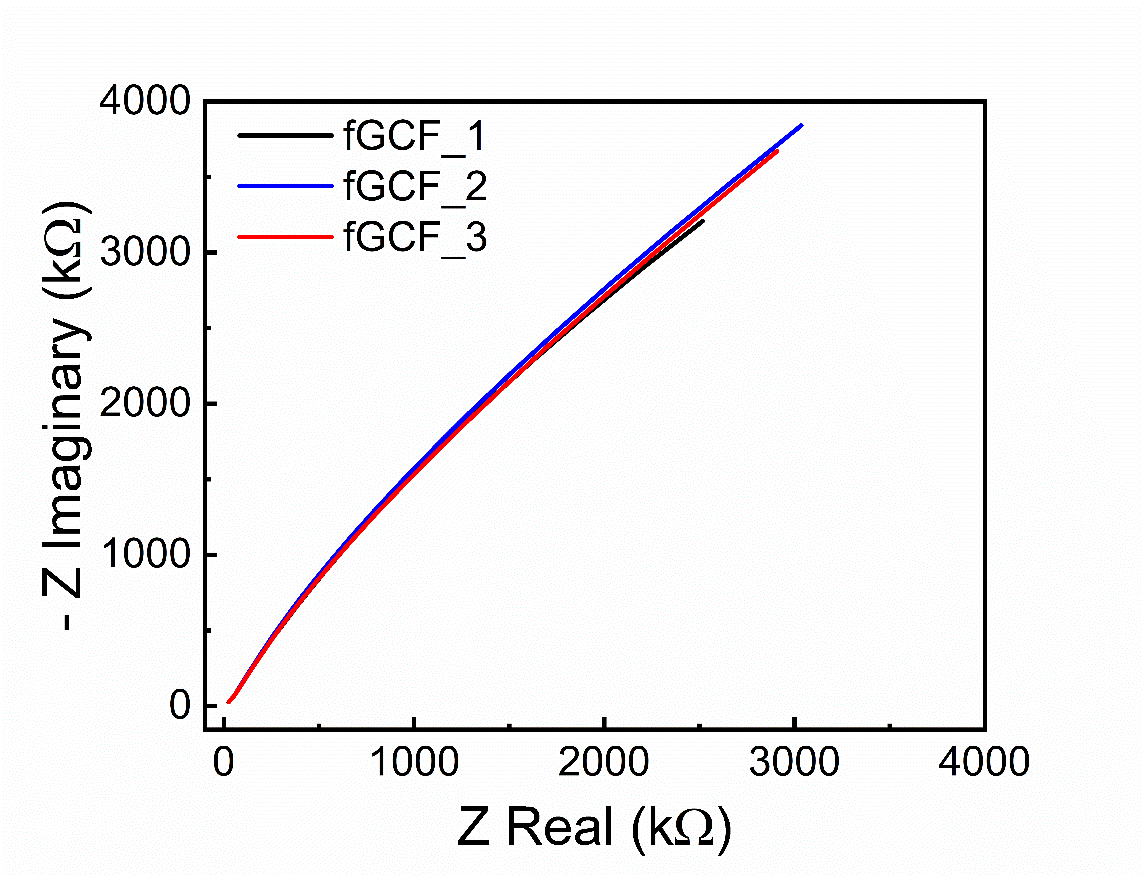

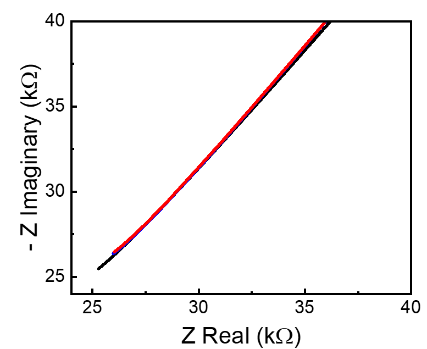


A

B

**Supplementary Figure 2. A)** Cyclic voltammograms (CVs) at different scan rates and **B)** Nyquist plots of full glassy carbon fibers (fGCFs) in the presence of 5 mM K_3_[Fe(CN)_6_], 5 mM K_4_[Fe(CN)_6_], a well-known redox couple used to investigate the electron transfer kinetics of sensor surfaces, and 1 M KCl as the supporting electrolyte.





**Supplementary Figure 3.** Representative Square Wave Voltammetry (SWV) plots without baseline subtraction obtained from fGCF conducted in 1x PBS in 50 nM -1000 nM concentration range.

References

1. Nimbalkar S, Castagnola E, Balasubramani A, Scarpellini A, Samejima S, Khorasani A, et al. Ultra-capacitive carbon neural probe allows simultaneous long-term electrical stimulations and high-resolution neurotransmitter detection. Scientific reports. 2018;8(1):6958.
